# Practical Predefined-Time Sliding-Mode Adaptive Resilient Control for PMSM Cyber–Physical Systems

**DOI:** 10.3390/s25237380

**Published:** 2025-12-04

**Authors:** Zhenzhong Wang, Shu Zhang, Yun Jiang, Chunwu Yin

**Affiliations:** 1 Advanced Institute of Information Technology, Peking University, Hangzhou 311200, China; 2 School of Computer Science, Peking University, Beijing 100871, China; 3 School of Information and Control Engineering, Xi’an University of Architecture and Technology, Xi’an 710055, China

**Keywords:** cyber physical systems, cyber-attacks, resilient control, predefined time control, PMSM

## Abstract

The permanent magnet synchronous motor (PMSM) is extensively utilized in the power drive systems of Cyber–Physical Systems (CPSs). In scenarios where control signals are subjected to malicious attacks within the network, ensuring that the PMSM achieves its designated speed within a specified timeframe serves as a critical metric for evaluating the efficacy of security control strategies in networked systems. To address practical challenges arising from updates to controlled objects at the physical layer and limitations of control layer algorithms—wherein convergence time for system trajectory tracking errors (TTEors) may extend indefinitely—we have developed a novel resilient control algorithm with predefined-time convergence (PreTC) tailored for uncertain PMSMs susceptible to cyber threats. Firstly, we introduce an innovative Lyapunov stability criterion characterized by an adjustable gain reaching law alongside PreTC. Following this, we design an SMS (SMS) that incorporates PreTC and employ an extreme learning machine (ELM) to facilitate real-time identification of both physical layer models and malicious cyber-attacks. A sliding-mode adaptive resilient controller devoid of explicit physical model information is proposed for CPSs, with Lyapunov stability theory substantiating the system’s predefined-time (PDT) stability. This significantly enhances resilience against malicious cyber-attacks and other uncertainties. Finally, comparative simulations involving four distinct resilient control algorithms demonstrate that our proposed algorithm not only guarantees predetermined convergence times but also exhibits robust resistance to cyber-attacks, parameter perturbations, and external disturbances—notably achieving a motor speed tracking error accuracy of 0.008. These findings validate the superior robustness and effectiveness of our control algorithm against malicious cyber threats.

## 1. Introduction

Cyber-physical systems (CPSs) represent an advanced intelligent framework capable of seamlessly integrating computation, communication, and control mechanisms. By fusing human–computer interaction capabilities with real-time network communication technologies, the CPS enables secure and reliable management of remote physical entities [1]. This innovative technology has found widespread applications across multiple domains, including the Internet of Things (IoT), smart homes, and aerospace engineering. The evolution of CPS is characterized by continuous enhancements in intelligence, flexibility, scalability, and collaboration. Consequently, in practical engineering applications, CPS must exhibit high reliability, safety, control accuracy, as well as exceptional perception and autonomous control capabilities. Permanent magnet synchronous motors (PMSMs), renowned for their wide speed regulation range and high control precision, are frequently employed as driving devices for remote operations within CPS frameworks. However, technological advancements and changes in application environments often necessitate the update or replacement of driving motors, while also introducing challenges such as malicious cyber-attacks, prescribed-time stability requirements, and unforeseen disturbances. These issues may adversely affect the universality, accuracy, stability, and transient performance of PMSM-based CPS. Therefore, effectively addressing the universality, stability, and accuracy challenges of PMSM-CPS holds significant importance in industrial automation and high-performance control applications.

In recent years, research on defenses against integrity attacks has encompassed a diverse range of systems and technologies. Regarding cyber-attack detection techniques, researchers have adopted hybrid approaches combining nonlinear observers, Chi-square detectors, mixed-integer nonlinear programming, and state estimation to achieve attack detection. Within the realm of wireless sensor networks, investigators have explored malicious information injection and state estimation algorithms in Krein space. While these algorithms offer the advantage of addressing unforeseen attacks, their detection efficiency remains unstable [2]. Reference [3] proposed a minimum defense budget state estimation algorithm for power systems vulnerable to false data injection (FDI) attacks, relaxing the assumption that “certain meter measurements can fully resist such attacks.” Reference [4] employed a Chi-square detector combined with the Cosine Similarity Matching (CSM) method for attack detection, demonstrating robust performance in identifying both FDI attacks and other attack types. Reference [5] introduced a decentralized homomorphic computing paradigm for system state estimation and designed a centralized FDI detector specifically tailored to identify FDI attacks. Despite their utility, these methods face challenges such as reliance on numerous attack model assumptions and the need for improved accuracy. Furthermore, these approaches primarily focus on detecting attacks rather than ensuring system stability in the presence of attacks. To address this gap, scholars have proposed resilient control strategies that enable CPS to maintain favorable steady-state performance even when confronting cyber-attacks. To solve the resilient control problem of linear CPS under FDI attacks and process interference, Reference [6] proposed a control strategy integrating an attack-resilient state observer with a control gain-switching mechanism, whose effectiveness was validated through anti-attack simulations on a linearized reduced-order aircraft system. Reference [7] combined a Butterworth low-pass filter with radial basis function neural networks to construct a neural network observer for estimating unmeasurable states, and designed an adaptive event-triggered neural network resilient control method for CPS to ensure system stability. Additionally, Reference [8] developed a distributed observer to stabilize the estimation error system and proposed an algorithm capable of exposing attacker behavior and identifying tampered sensors. Reference [9] designed a sliding-mode resilient control strategy for network systems subjected to DoS attacks, effectively enhancing the system’s ability to resist DoS attacks. Although these algorithms improve CPS’s resilience against various cyber-attacks, they rely heavily on physical layer model information, failing to meet the application requirements of rapid iteration and update of current physical layer intelligent terminals [10]. Therefore, establishing resilient control strategies that minimally depend on or are independent of physical layer model information is more aligned with technological iteration needs. However, model-independent resilient control strategies are currently scarce and remain in the early stages of research.

Ensuring the stability of cyber-attacked CPS within a specified timeframe is a critical metric for evaluating the effectiveness of cyber system security control strategies. Nevertheless, existing resilient control strategies predominantly rely on asymptotic stability, often leading to infinite convergence time for CPS. To enhance the convergence speed of controlled systems, researchers have proposed finite-time control algorithms. For instance, Zhang W et al. [11] introduced a generalized super-twisting observer to estimate the rotor position and speed of PMSMs, endowing the speed controller with finite-time convergence capability. Additionally, Chen Z et al. [12] developed a finite-time control algorithm based on the backstepping method, considering the dynamic performance constraints of angular position tracking errors. While both algorithms guarantee finite-time convergence of motor systems, they neglect parameter uncertainties and external disturbances, compromising their robustness. Sliding-mode control (SMC) exhibits strong robustness, and its combination with finite-time control methods has been successfully applied to trajectory tracking in PMSM systems. Li S et al. [13] proposed a non-singular terminal sliding-mode surface (SMS) and designed a speed controller based on this surface, achieving system state convergence to the sliding-mode surface and equilibrium point within finite time. Feng Y et al. [14] designed a disturbance observer for finite-time convergent estimation of uncertainty components in PMSMs, and developed an observer-based terminal SMC to ensure that the PMSM position converges to the target position within finite time. Echreshavi, Z [15,16,17] approached the problem from the perspective of event-triggered control, designing a series of sliding-mode control strategies for uncertain nonlinear systems that not only enhance system robustness but also effectively reduce control input energy consumption. Despite the fact that such finite-time control algorithms improve the convergence speed of PMSM systems, a critical limitation is that the upper bound of convergence time depends on both initial states and controller parameters, rendering it unpredictable. Consequently, researchers have developed fixed-time controllers, whose convergence time upper bound is solely determined by control parameters. For example, Reference [18] designed a fixed-time convergence controller and applied it to wheeled robots with control dead zones; Xu P [19] proposed a fixed-time stability controller for dynamic positioning ships under unknown disturbances; Wang L [20] applied fixed-time control algorithms to PMSMs by designing a non-singular fast terminal sliding-mode controller (TSMC) with a fixed-time convergence law. It is important to note that the maximum value of this fixed-time convergence algorithm has specific limitations, as it directly influences system stability through its dependence on control parameters. Subsequently, researchers designed Predefined-Time Sliding-Mode Controllers (PDT SMCs) for various uncertain systems [21,22,23]. Although the convergence time of these PDT controllers can be preset, they are all designed based on fixed-time convergence stability criteria. Munoz-Vazquez A. J. [24] proposed a PDT stability criterion where the upper bound of convergence time is independent of both initial states and control parameters. However, the parameters of the proposed stability criterion lack adjustability, preventing performance tuning of the controlled system. On the other hand, sliding-mode control consists of an approaching phase and a sliding phase. Existing PDT control strategies can only guarantee that the SMS converges to zero within the PDT, but the tracking error at this point remains unstable. The tracking error within the SMS still needs to reach the origin through the sliding phase to achieve stability. Since the SMSs in existing PDT SMC strategies do not possess PDT convergence characteristics, the SMC cannot ensure that the trajectory tracking error of CPS exhibits PDT convergence.

By analyzing the above discussion, the current security control strategies for cyber–physical systems (CPSs) exhibit certain deficiencies:

(1) Most existing security control strategies rely on the model information of the controlled object at the physical layer. However, when changes occur in the intelligent terminals of CPS, this can lead to control failures, rendering model-based control strategies insufficiently universal. Moreover, such strategies fail to meet cost-saving requirements during CPS update iterations. Therefore, there is an urgent need to develop an adaptive control strategy that does not depend on the model information of the controlled object. Nevertheless, current research on model-free and disturbance-independent control strategies for CPS remains limited.
(2) Stability criteria for both finite-time convergence and fixed-time convergence have been proposed for NLS. However, practical NLS control faces limitations: the upper bound (UB) of convergence time in finite/fixed-time control theory depends on initial values and control parameters, resulting in a non-predetermined UB of convergence time. Although A. J. Muñoz–Vázquez proposed a PreTC stabilization criterion for NLS, allowing arbitrary setting of convergence time, this criterion designs a controller with fixed gains in the reaching law. Consequently, parameter adjustment for dynamic performance optimization is not feasible.
(3) The application of SMC as a safety control strategy for CPS is prevalent. However, existing sliding-mode security control strategies for CPS can only ensure asymptotic convergence and provide no guarantees regarding the convergence of attacked CPS within a PDT. The PDT in SMC consists of two phases: the time required for the TTEor to reach the SMS and the time for the TTEor to converge to the equilibrium point. Current SMSs lack the capability to predefine convergence time, making it impossible to set an UB on the convergence time of TTEor after reaching the SMS. Consequently, the actual convergence time of the TTEor cannot be predetermined.

In light of the aforementioned deficiencies in CPS security controls, this paper aims to make the following improvements:

(1) Although A. J. Muñoz–Vázquez proposed a PreTC stabilization criterion for NLSs, the controller gains are fixed, precluding adjustments to dynamic performance. This paper extends the stability criterion proposed by A. J. Muñoz–Vázquez to enhance PreTC theory. Specifically, controllers with adjustable gains in the reaching law are designed, enabling dynamic performance optimization of the controlled object through gain-tuning.
(2) A novel SMS is proposed to contain the TTEor, allowing the UB of the TTEor‘s convergence time to be arbitrarily set according to engineering requirements. The controller designed based on this improved SMS, combined with the PreTC criterion proposed in this paper, ensures that the TTEor of CPS converges within the specified UB of convergence time.
(3) The proposed approach employs an extreme learning machine (ELM) to estimate the system model and detect malicious cyber-attacks in real-time, using the TTEor as input information. By integrating the proposed SMS with the PreTC stabilization criterion, this paper develops a novel sliding-mode adaptive controller with a concise structure. This controller guarantees that the TTEor of CPS converges within a predetermined timeframe, thereby enhancing the universality and attack resilience of CPS security controllers.

The paper is organized section-wise: Section 2 describes the CPS, which includes the model of the PMSM in the physical layer, the attack model in the network layer, and the control objectives. Section 3 introduces a novel PreTC stability theory along with an innovative SMS incorporating PreTC. Section 4 details the primary design of a PDT sliding-mode adaptive resilient controller based on ELM and discusses its stability. Numerical simulations presented in Section 5 validate the proposed control strategy, focusing primarily on dynamic performance under malicious cyber-attacks. Comparative simulation analyses are conducted under varying predefined convergence times and different controllers. Finally, our study concludes in Section 6.

## 2. Description of Cyber–Physical Systems

**Figure 1:**
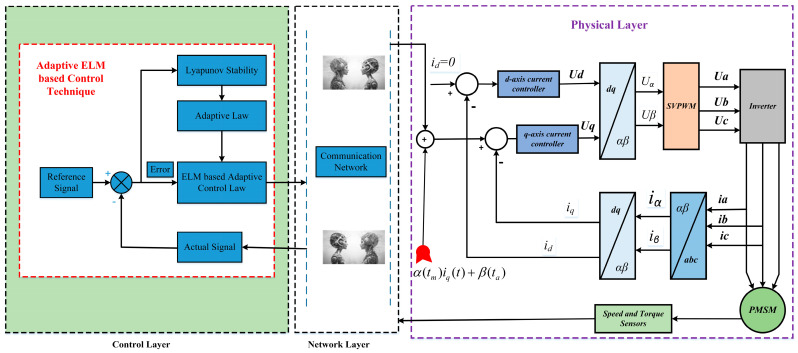
Structural diagram of cyber-attacked CPS model.

The PMSM offers numerous advantages, including a simple structure, low moment of inertia, wide speed range, and high efficiency. It is widely used in high-precision numerical control machines, robotics, and the aerospace industry. This paper addresses the security control problem of CPS where the network layer is subjected to malicious threat signals and the physical layer consists of a PMSM. An adaptive resilient SMC that does not rely on models and is disturbance-independent has been proposed. The overall structure of the CPS is illustrated in Figure 1 below.

### 2.1. PMSM’s Model of the Physical Layer

In physical layer, the PMSM’s voltage equation in CPS is [25].

(1) $$\left\{\begin{array}{l} {\dot{i}}_{d}=(u_{d}-Ri_{d}+\omega_{e}L_{q}i_{q})/L\\ {\dot{i}}_{q}=(u_{q}-Ri_{q}-\omega_{e}(L_{d}i_{d}+\psi_{f}))/L \end{array}\right.$$

From above expression, the $u_{d},u_{q},i_{d},i_{q}$ denote the stator voltage and current in the d-axis and q-axis, correspondingly; R denotes the stator resistance; L denotes the synchronous inductance, and $L_{d},\;L_{q}$ denote the inductance in the d-axis and q-axis; $\psi_{f}$ is the magnetic chain of the PMSM; and $\omega_{e}$ denotes the electrical angular velocity.

The mechanical equations of motion for PMSM can be described as [26].

(2) $$J{\dot{\omega }}_{r}=T_{e}-B\omega_{r}-T_{L}$$

The parameter $\omega_{r}$ signifies the mechanical angular velocity of the rotor; J is the inertia moment; $T_{L}$ is the load torque; B is the damping coefficient; $T_{e}$ denotes an electromagnetic torque and its mathematical model is given as the following:

(3) $$T_{e}=1.5p\psi_{f}i_{q}$$

where p is the number of poles of the motor and satisfies $e_{e}=p\omega_{r}$. Substituting Equation (3) into Equation (2) and letting $\chi =\left(1.5p\psi_{f}\right)/J$, η=B/J, γ=1/J, It is rewritten as:

(4) $${\dot{\omega }}_{r}=\chi i_{q}-\eta \omega_{r}-\gamma T_{L}$$

### 2.2. Attack Model of the Network Layer

In the network layer, the open nature of communication networks renders CPS vulnerable to malicious cyber threats during remote real-time control implementation, such as denial-of-service (DoS) and false data injection (FDI) attacks.

A DoS attack is a typical malicious assault that attempts to flood the information transmission channels in a network with massive traffic, thereby disrupting network signal transmission. In remotely operated systems, DoS attacks degrade the control commands sent to the control layer by consuming network-layer resources, ultimately affecting the control performance of the physical layer. This type of model under a DoS attack can be expressed as follows:

(5) $$i_{q\text{\_}\mathrm{attack}}(t)=\kappa_{\mathrm{Dos}}(t_{m})i_{q}(t)$$

where $\kappa_{\mathrm{Dos}}(t_{m})>0$ is the weakening/increasing ratio; $t_{m}$ is the initial attack time.

FDI attacks represent a type of cyber intrusion that leverages forged data to manipulate the allocation of resources within target computer networks. Within remote control frameworks, FDI introduces deceptive data into network-level control signals. This interference has a cascading effect on the physical layer’s control effectiveness. An FDI attack within the network layer is

(6) $$i_{q\text{\_}\mathrm{attack}}(t)=i_{q}(t)+\lambda_{\mathrm{FDI}}(t_{a})$$

where $\lambda_{\mathrm{FDI}}(t_{a})$ is the deviation in FDI attacks; $t_{a}$ is the initial attack time.

There are various types of cyber-attacks, but all attacks are aimed at weakening/increasing control inputs or biasing control input signals, so the control input becomes the following:

(7) $$i_{q\text{\_}\mathrm{attack}}(t)=\kappa_{\mathrm{Dos}}(t_{m})i_{q}(t)+\lambda_{\mathrm{FDI}}(t_{a})$$

where $i_{q\text{\_}\mathrm{attack}}(t)$ represents input current after cyber-attack, $\kappa_{\mathrm{Dos}}(t_{m})\in (0,\infty )$ is the multiplicative attack threat, and $\lambda_{\mathrm{FDI}}(t_{a})$ is the additive attack threat.

Due to the unknown and uncertain nature of $\kappa_{\mathrm{Dos}}(t_{m})$ and $\lambda_{\mathrm{FDI}}(t_{a})$, the cyber-attack (7) can be modified as

(8) $$i_{q\text{\_}\mathrm{attack}}(t)=i_{q}(t)+(\kappa_{\mathrm{Dos}}-1)i_{q}(t)+\lambda_{\mathrm{FDI}}$$

### 2.3. Control Objective

There are parametric perturbations during the motor rotation process, which lead to unknown disturbances Δχ, Δη, Δγ in the system parameters χ, η, γ, respectively, and denote known mode information $g=-\eta \omega_{r}-\gamma T_{L}$, and unknown $d=\Delta \chi (\alpha i_{q}+\beta )+\chi ((\alpha -1)i_{q}+\beta )-\Delta \eta \omega_{r}$ $-\Delta \gamma T_{L}$; then the uncertain PMSM’s mechanical equation under cyber-attack is simplified as

(9) $${\dot{\omega }}_{r}=\chi i_{q}+g+d$$

Let the desired trajectory of the mechanical angular velocity be $\omega_{r}^{*}(t)$. Denote the angular velocity TTEor as

(10) $$e(t)=\omega_{r}^{*}(t)-\omega_{r}(t)$$

The objective of the CPS is to design a reference input $i_{q}^{*}$ for the q-axis current in PMSM that accounts for malicious cyber-attacks and parameter perturbations. This will enable the angular velocity TTEor e(t) to converge to zero within the PDT $T_{s}$.

## 3. Novel PDT Convergence Stability Norm

To design a PreTC resilient controller for a PMSM CPS, a novel PreTC Lyapunov stability criterion is proposed and proved firstly.

Consider a nonlinear system (NLS) described by the following dynamical equation:

(11) $$\dot{x}=f(x),\;x(0)=x_{0}$$

where $x\in {ℜ}^{n}$ denotes the system’s state; function $f:{ℜ}^{n}\to {ℜ}^{n}$ represents the system dynamics; it is a smooth and differentiable function. $x(0)=x_{0}$ are the initial conditions.

**Theorem** **1.** *For NLS (11), and for arbitrary PDT* $T_{s2}>0$*, if there exists a radial unbounded and positive definite Lyapunov function* σ(x(t)) *which satisfies*

(12) $$\dot{\sigma }(x)\leq \frac{-\pi }{2p_{2}T_{s2}\sqrt{a_{2}b_{2}}}[a_{2}\sigma {\left(x\right)}^{1-p_{2}}+b_{2}\sigma^{1+p_{2}}{\left(x\right)}^{1-p_{2}}]$$

 *among them, and the parameters are* $0<p_{2}<1,\;a_{2}>0,\;b_{2}>0$*, the NLS (11) is global PDT stability, and the time* $t_{s}$ *of the state variable converges till the equilibrium point satisfies*

$$t_{s}=\frac{2T_{s2}}{\pi }\arctan[\sqrt{b_{2}/a_{2}}\sigma {\left(x_{0}\right)}^{p_{2}}]<T_{s2}$$

**Proof.**  According to $\dot{\sigma }(x(t))\leq -\frac{\pi }{2p_{2}T_{s2}\sqrt{a_{2}b_{2}}}[a_{2}\sigma {\left(x\right)}^{1-p_{2}}+b_{2}\sigma {\left(x\right)}^{1+p_{2}}]$, let $\dot{\sigma }(x)=\frac{\pi [a_{2}\sigma {\left(x\right)}^{1-p_{2}}+b_{2}\sigma {\left(x\right)}^{1+p_{2}}]}{-2p_{2}T_{s2}\sqrt{a_{2}b_{2}}}-\delta$, where δ>0, then

(13) $$\frac{d\sigma (x)}{dt}=\frac{-\pi a_{2}\sigma {\left(x\right)}^{1-p_{2}}}{2p_{2}T_{s2}\sqrt{a_{2}b_{2}}}[1+\frac{b_{2}}{a_{2}}\sigma {\left(x\right)}^{2p_{2}}+\frac{2p_{2}T_{s2}\sqrt{a_{2}b_{2}}}{\pi a_{2}\sigma {\left(x\right)}^{1-p_{2}}}\delta ]$$

After transforming Equation (13) and gathering together the differential, the following equation is obtained.

(14) $$\frac{\pi dt}{2T_{s2}}=\frac{-d[\sqrt{b_{2}/a_{2}}\sigma {\left(x\right)}^{p_{2}}]}{1+{\left[\sqrt{b_{2}/a_{2}}\sigma {\left(x\right)}^{p_{2}}\right]}^{2}+\frac{2p_{2}T_{s2}\sqrt{a_{2}b_{2}}}{\pi a_{2}\sigma {\left(x\right)}^{1-p_{2}}}\delta }$$

Suppose that $\sigma (x(t_{s}))=0$ at $t_{s}$, integrating both sides of Equation (14). Since σ(x)≥0, δ≥0, then $\frac{2p_{2}T_{s2}\sqrt{a_{2}b_{2}}}{\pi a_{2}\sigma {\left(x\right)}^{1-p_{2}}}\delta \geq 0$, which provides

$$\begin{array}{cc} \int_{0}^{t_{s}}\frac{\pi }{2T_{s2}}dt & =-\int_{\sigma (x_{0})}^{\sigma (x(t_{s}))}\frac{d(\sqrt{b_{2}/a_{2}}\sigma {\left(x\right)}^{p_{2}})}{1+{\left(\sqrt{b_{2}/a_{2}}\sigma {\left(x\right)}^{p_{2}}\right)}^{2}+\frac{2p_{2}T_{s2}\sqrt{a_{2}b_{2}}}{\pi a_{2}\sigma {\left(x\right)}^{1-p_{2}}}\delta }=\int_{0}^{\sigma (x_{0})}\frac{d(\sqrt{b_{2}/a_{2}}\sigma {\left(x\right)}^{p_{2}})}{1+{\left(\sqrt{b_{2}/a_{2}}\sigma {\left(x\right)}^{p_{2}}\right)}^{2}+\frac{2p_{2}T_{s2}\sqrt{a_{2}b_{2}}}{\pi a_{2}\sigma {\left(x\right)}^{1-p_{2}}}\delta }\\ & \leq \int_{0}^{\sigma (x_{0})}\frac{1}{1+{\left(\sqrt{b_{2}/a_{2}}\sigma {\left(x\right)}^{p_{2}}\right)}^{2}}d(\sqrt{b_{2}/a_{2}}\sigma {\left(x\right)}^{p_{2}})\Rightarrow t_{s}\leq \frac{2T_{s2}}{\pi }\arctan[\sqrt{b_{2}/a_{2}}\sigma {\left(x_{0}\right)}^{p_{2}}]\leq T_{s2} \end{array}$$

The above proof demonstrates that the convergence time of the nonlinear system (NLS) in (11) satisfies $t_{s}<T_{s2}$. This indicates that the state variable can converge to zero within the PDT $T_{s2}$, where the UB of the convergence time is independent of both the initial state and control parameters. Thus, the system’s convergence time can be preset arbitrarily. □

**Remark** **1.** *The stability criterion, and the upper of convergence time are shown in Table 1. Compared with the Lyapunov stability theory of* $\dot{\sigma }(x)\leq \frac{-\pi (\sigma {\left(x\right)}^{0.5}+\sigma {\left(x\right)}^{1.5})}{T_{s2}}$ *proposed in Reference [24], the novel PreTC Lyapunov stability criterion proposed in this paper incorporates parameters* $a_{2}>0,\;b_{2}>0$*. These parameters enable adjustability of the reaching law gain in the controller designed based on the given stability criterion, thereby facilitating the tuning of the controlled system’s dynamic performance. If* $a_{2}=b_{2}=1,\;p_{2}=0.5$ *are satisfied, the proposed Lyapunov stability criterion is equivalent to that presented in Reference [24]. For the NLS in (11), if the Lyapunov function is* $\sigma =0.5x^{Τ}x$*, the PDT controller derived based on the stability criterion in Reference [24] is* $u=-f(x)-\frac{\pi }{T_{s2}}(sign(x)+x^{2}\mathrm{sign}(x))$*. However, based on the novel stability criterion proposed in this paper, the controller is* $u=-f(x)-\frac{\pi (a_{2}x^{1-p_{2}}+b_{2}x^{1+p_{2}})}{2p_{2}T_{s2}\sqrt{a_{2}b_{2}}}$*. By comparing these two controllers, it can be observed that the gain of the latter is adjustable.*

**Remark** **2.** *Current controllers designed based on finite-time/fixed-time control theory merely ensure that the TTEor of the NLS converges within a finite time. However, the UB of this convergence time depends on both the initial state values and controller parameters, and thus cannot be predetermined. In contrast, by leveraging the novel Lyapunov stability criterion proposed in this paper, we achieve a predefined convergence time* $T_{s2}$ *for the system’s TTEor to converge to zero. This guarantees that an explicit upper limit for the overall convergence time can be established in advance.*

**Remark** **3.** *Building upon the PreTC stability criterion proposed in this paper, control techniques such as SMC, adaptive control, backstepping control, and prescribed performance control can be integrated to design corresponding PDT controllers for arbitrary uncertain NLSs.*

## 4. Sliding-Mode Controller Design

### 4.1. Controller Desigen

*Step 1:* SMS design

To fully exploit the robustness advantage of SMClers, and ensure that the TTEor e(t) can converge within a PDT $T_{s}$, the following sliding-mode surface (SMS) with a PreTC is designed:

(15) $$S=e(t)+\frac{\pi }{2p_{1}T_{s1}\sqrt{a_{1}b_{1}}}(a_{1}\varphi (t)+b_{1}\xi^{1+p_{1}}(t))$$

where the parameters satisfy $0<p_{1}<1,\;a_{1}>0,\;b_{1}>0$; $T_{s1}$ is the PDT; $\xi (t)=\int_{0}^{t}e(\tau )d\tau$. $0<\delta_{\xi }<1$, $l_{2},l_{3}>0$ are parameters to be designed; $l_{1}=\delta_{\xi }^{-p_{1}}-l_{2}\delta_{\xi }-l_{3}\delta_{\xi }^{2}$; φ(t) is defined as

(16) $$\varphi (t)=\left\{\begin{array}{l} \xi^{\left[1-p_{1}\right]},\;\;\;\;\;\;\;\;\;\;\;\;\;\;\;\;\;|\xi |>\delta_{\xi }\\ l_{1}\xi +l_{2}\xi^{\left[2\right]}+l_{3}\xi^{\left[3\right]},\;\;|\xi |\leq \delta_{\xi } \end{array}\right.$$

**Remark** **4.** *If* ξ>0*,* $\underset{\xi \to \delta_{\xi }^{+}}{\lim}\varphi =\underset{\xi \to \delta_{\xi }^{+}}{\lim}\xi^{\left[1-p_{1}\right]}=\delta_{\xi }^{1-p_{1}}$*,*$\underset{\xi \to \delta_{\xi }^{-}}{\lim}\varphi =l_{1}\delta_{\xi }+l_{2}\delta_{\xi }^{2}+l_{3}\delta_{\xi }^{3}=\delta_{\xi }^{1-p_{1}}$*, then* φ(t) *is continuous at* $\xi =\delta_{\xi }$*. When* ξ<0*,* $\underset{\xi \to -\delta_{\xi }^{+}}{\lim}\varphi =\underset{\xi \to -\delta_{\xi }^{+}}{\lim}\xi^{\left[1-p_{1}\right]}=-\delta_{\xi }^{1-p_{1}},\underset{\xi \to -\delta_{\xi }^{-}}{\lim}\varphi ==-l_{1}\delta_{\xi }-l_{2}\delta_{\xi }^{2}-l_{3}e_{\xi }^{3}=-\delta_{\xi }^{1-p_{1}}$*; therefore,* φ(t) *is continuous at* $\xi =-\delta_{\xi }$*. Taken together, the function* φ(t) *is continuous, which solves the problem of control input jumping caused by discontinuity at segmentation points in traditional functions.*

According Equation (16)*,* we have

(17) $$\dot{\varphi }(t)=\left\{\begin{array}{l} (1-p_{1})\xi^{\left[-p_{1}\right]},\;\;\;\;\;\;\;\;\;\;\;\;\;|\xi |>\delta_{\xi }\\ (l_{1}+2l_{2}\xi^{\left[1\right]}+3l_{3}\xi^{\left[2\right]}),\;\;\;|\xi |\leq \delta_{\xi } \end{array}\right.$$

**Figure 2:**
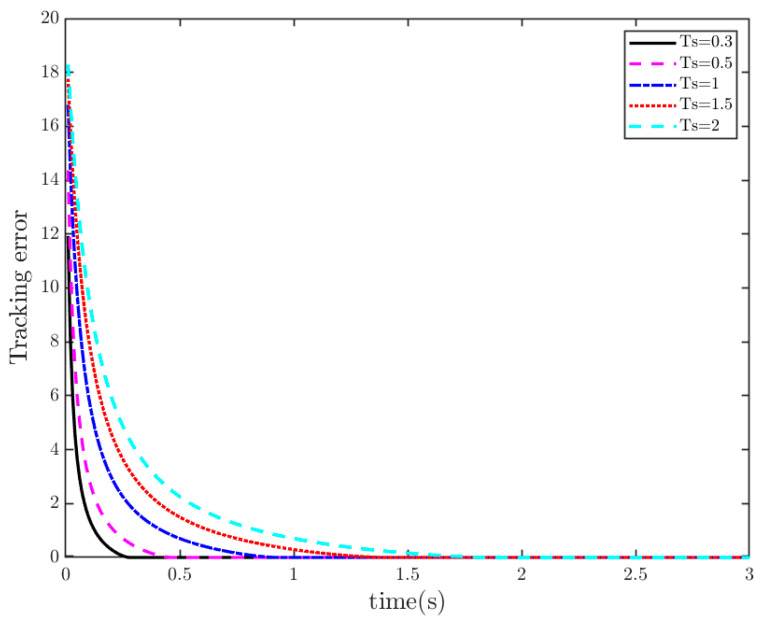
TTEor for different predefined convergence times.

**Remark** **5.** *To confirm the PreTC characteristic of TTEor within SMS, we assume that TTEor reaches the SMS (designated as* S=0*) and set the convergence times as* $T_{s1}=0.3\;s,\;0.5\;s,\;1\;s,\;1.5\;s,\;2\;s$*. The simulation is shown in Figure 2. It shows that the actual convergence times are persistently shorter than the PDT, as the TTEor grasps the SMS. This implies that the TTEor situated on SMS (15) has the property of PDT convergence.*

*Step 2:* Design Compensator Based on ELM

Derive the SMS S, then

(18) $$\begin{array}{l} \dot{S}(t)=\frac{\pi (b_{1}(1+p_{1})\xi^{p_{1}}(t)+a_{1}\dot{\varphi }(t))e(t)}{2p_{1}T_{s1}\sqrt{a_{1}b_{1}}}+\dot{e}(t)\\ =\omega_{r}^{*}(t)-\chi i_{q}(t)-g(t)-d(t)+\frac{\pi (b_{1}(1+p_{1})\xi^{p_{1}}(t)+a_{1}\dot{\varphi }(t))e(t)}{2p_{1}T_{s1}\sqrt{a_{1}b_{1}}} \end{array}$$

**Figure 3:**
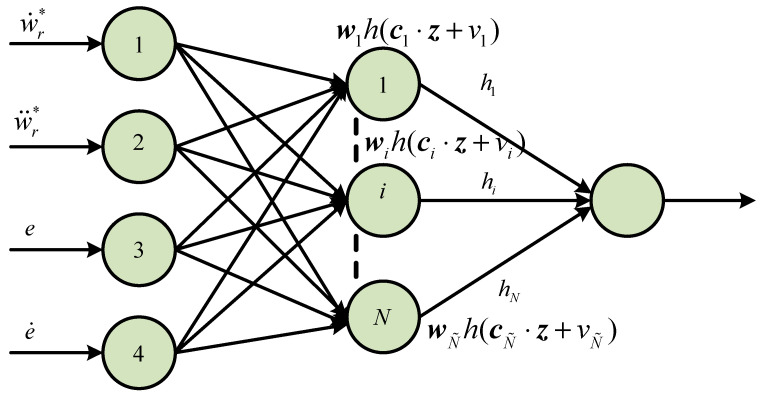
Diagram of SLFN structure.

Let G=g+d represent the encapsulated component, which can be estimated with ELM [26]. The ELM is regarded as single hidden-layer feed-forward neural networks (SLFNNs), and the structural of SLFNs is shown in Figure 3.

The input of the SLFN is $z={\left[e,\xi \right]}^{Τ}$, $\tilde{N}$ is the nodes, and h(x) is the activation function. Through continuous learning and training, it is possible to obtain the optimal weight vector $w^{*}$ of SLFN in order to approximate the encapsulated component G.

(19) $$G=\sum_{i=1}^{\tilde{N}}w_{i}^{*}h_{i}(c_{i}z,v_{i})=w^{*}h(z)$$

where $c_{i}={\left[c_{i1},c_{i2},\ldots c_{in}\right]}^{Τ}\in R^{n}$ is the internal connection weight to be designed, $v_{i}$ is the node threshold to be designed, and $w_{i}={\left[w_{i1},w_{i2},\ldots w_{im}\right]}^{Τ}\in R^{m}$ is the external connection weight.

SLFNs need to learn and adjust their weight vectors $c_{i}={\left[c_{i1},c_{i2},\ldots c_{in}\right]}^{Τ}$, $w_{i}={\left[w_{i1},w_{i2},\ldots w_{im}\right]}^{Τ}$, and bias values $v_{i}$, which can result in a large amount of computation and slow computation speed. To make SLFNs easier to compute, the ELM is proposed by Huang G B [27]. In the ELM, the connection weights $c_{i}$ and the node thresholds $v_{i}$ have been arbitrarily created. $w_{i}$ is the outer weight vector to be recognized. $h(z)={\left(h_{1}(c_{1}z,v_{1}),\ldots ,h_{\tilde{N}}(c_{\tilde{N}}z,v_{\tilde{N}})\right)}^{Τ}$. The estimation of $w^{*}$ is obtained indirectly in practical engineering applications and cannot be approximated as G precisely. The value of $\hat{G}$ is calculated as

(20) $$\hat{G}=h(z)\hat{w}=\sum_{i=1}^{\tilde{N}}{\hat{w}}_{i}h_{i}(c_{i}z,v_{i})$$

In the above formula, the ${\hat{w}}_{i}$ signifies an estimation of the optimal value $w_{i}^{*}$, and the designed adaptive law is as follows:

(21) $$\dot{\hat{w}}=-\gamma hS$$

*Step 3:* Controller design.

We designed the following resilient controller:

(22) $$i_{q}^{*}=i_{a}+i_{b}$$

where

(23) $$\begin{array}{l} i_{a}=\frac{1}{\chi }\left(\omega_{r}^{*}-\hat{G}+\frac{\pi (a_{1}\dot{\varphi }(t)+b_{1}(1+p_{1})\xi^{p_{1}})}{2p_{1}T_{s1}\sqrt{a_{1}b_{1}}}e\right)\\ i_{b}=\frac{\pi }{\chi p_{2}T_{s2}\sqrt{a_{2}b_{2}}}(a_{2}S^{1-p_{2}}+b_{2}S^{1+p_{2}}) \end{array}$$

where constant $a_{i}>0,\;b_{i}>0,\;0<p_{i}<1$.

**Remark** **6.** *The controller proposed in this paper exclusively utilizes the angular velocity tracking error information of the PMSM, without relying on any physical layer model information. This controller exhibits excellent universality, as it does not require explicit knowledge of the PMSM’s detailed mechanical model or external disturbances.*

### 4.2. Stability Analysis

**Theorem** **2.** *For arbitrary PDT* $T_{s1}>0,\;T_{s2}>0$*, when the q-axis current reference input* $i_{q}^{*}$ *of the PMSM (9) in the physical layer is (22), the angular velocity TTEor of the PMSM will converge to the SMS (15) within the PDT* $T_{s2}$*, and the upper-bounded angular velocity TTEor’s convergence time is *$T_{s}=T_{s1}+T_{s2}$.

**Proof.**  Taking the Lyapunov candidate as $\sigma_{1}=0.5S^{2}$, we derive t to obtain

(24) $${\dot{\sigma }}_{1}=S\dot{S}=S\left(\frac{\pi (a_{1}\dot{\varphi }(t)+b_{1}(1+p_{1})\xi^{p_{1}})e}{2p_{1}T_{s1}\sqrt{a_{1}b_{1}}}+\dot{e}\right)=S\left(\omega_{r}^{*}-\chi i_{q}-G+\frac{\pi (a_{1}\dot{\varphi }(t)+b_{1}(1+p_{1})\xi^{p_{1}})e}{2p_{1}T_{s1}\sqrt{a_{1}b_{1}}}\right)$$

Noting $\tilde{w}=w^{*}-\hat{w}$, $\tilde{G}=G-\hat{G}$, construct the Lyapunov function as $\sigma_{2}=\sigma_{1}+0.5\gamma^{-1}tr({\tilde{w}}^{Τ}\tilde{w})$, then

(25) $${\dot{\sigma }}_{2}=\frac{-\pi (\bar{a}\sigma_{1}^{1-\bar{p}}+\bar{b}\sigma_{1}^{1+\bar{p}})}{2\bar{p}T_{s2}\sqrt{\bar{a}\bar{b}}}-S\tilde{G}-\frac{tr({\tilde{w}}^{Τ}\dot{\hat{w}})}{\gamma }=\frac{\pi (\bar{a}\sigma_{1}^{1-\bar{p}}+\bar{b}\sigma_{1}^{1+\bar{p}})}{-2\bar{p}T_{s2}\sqrt{\bar{a}\bar{b}}}-\frac{tr({\tilde{w}}^{Τ}(\gamma hS+\dot{\hat{w}}))}{\gamma }$$

where $\bar{a}=a_{2}2^{1-0.5p_{2}},\;\bar{b}=b_{2}2^{1+0.5p_{2}},\;\bar{p}=0.5p_{2}$. By substituting Equation (22) into Equation (25), we obtain

$${\dot{\sigma }}_{2}=-\frac{\pi (\bar{a}V_{1}^{1-\bar{p}}+\bar{b}V_{1}^{1+\bar{p}})}{2\bar{p}T_{s2}\sqrt{\bar{a}\bar{b}}}$$

According to Theorem 1, the SMS S(t) will converge in PDT $T_{s2}$, and the state variables will converge to the SMS in PDT $T_{s2}$.

When the SMS satisfies S(t)=0, then

(26) $$e(t)=-\frac{\pi }{2p_{1}T_{s1}\sqrt{a_{1}b_{1}}}(a_{1}\varphi (t)+b_{1}\xi^{1+p_{1}}(t))$$

Define the Lyapunov candidate as $\sigma (t)=0.5\xi^{2}$, then obtain the following:

(27) $$\begin{array}{l} \dot{\sigma }(t)=\xi (t)\dot{\xi }(t)=-\frac{\pi (b_{1}\xi^{2+p_{1}}(t)+a_{1}\xi (t)\dot{\varphi }(t))}{2p_{1}T_{s1}\sqrt{a_{1}b_{1}}}\\ =\left\{\begin{array}{cc} -\frac{\pi ({\bar{a}}_{1}\sigma^{1-\frac{p_{1}}{2}}(t)+{\bar{b}}_{1}\sigma^{1+\frac{p_{1}}{2}}(t))}{2{\bar{p}}_{1}T_{s1}\sqrt{{\bar{a}}_{1}{\bar{b}}_{1}}} & |\xi |>\delta_{\xi }\\ -\frac{\pi (a_{1}\xi ((l_{1}+2l_{2}\xi^{\left[1\right]}+3l_{3}\xi^{\left[2\right]}))+b_{1}\xi^{2+p_{1}}(t))}{2p_{1}T_{s1}\sqrt{a_{1}b_{1}}} & |\xi |\leq \delta_{\xi } \end{array}\right. \end{array}$$

where ${\bar{p}}_{1}=\frac{p_{1}}{2},\;{\bar{a}}_{1}=a_{1}2^{1-\frac{p_{1}}{2}},\;{\bar{b}}_{1}=b_{1}2^{1+\frac{p_{1}}{2}}$.

According to Theorem 1, it can be concluded that when $|\xi (t)|>\delta_{\xi }$, ξ(t) will converge within the PDT $T_{s1}$. From Equation (26), if ξ(t)=0, then e(τ)≡0, so if the SMS *(15)* satisfies S=0, then the TTEor e(t) will converge at PDT $T_{s1}$. When $|\xi |\leq \delta_{\xi }$, ξ(t) will asymptotically converge stably to zero.

Combining the above analyses, it verified that the angular velocity trajectory tracking error e(t) can converge to zero within the PDT $T_{s}=T_{s1}+T_{s2}$. □

**Remark** **7.** *The motion trajectory of SMC is mainly divided into two phases: Phase (1) is the phase where state variables at any initial position converge to the SMS. This phase ensures that the TTEor converges toward the SMS (rather than the SMS itself converging to zero), while the state variables themselves do not converge to zero. Phase (2) is the phase where the state variables on the SMS converge to the equilibrium point (zero). In this phase, the state variables essentially achieve convergence to zero. Compared with existing finite-time/fixed-time SMCs, the latter can only ensure that state variables converge to the SMS within finite/fixed time in the first phase, but fail to guarantee that the state variables in the second phase also converge within a finite/fixed time. Therefore, traditional finite-time/fixed-time SMC is not essentially finite-time convergent in nature. In addition, these control algorithms do not possess the characteristic of PreTC. The algorithm proposed in this paper achieves essential PreTC.*

## 5. Validation Analysis

### 5.1. Simulation Environment

To verify the effectiveness of the predefined-time sliding-mode adaptive resilient control (PTSMAC) strategy designed in this paper, numerical simulations were performed via the MATLAB 2018 platform, utilizing the parameters of the PMSM listed in Table 2.

The q-axis current input $i_{q}^{*}$ of the PMSM angular velocity dynamics Equation (9) is the output value of the PTSMAC, and voltage inputs of Equation (1) are the PI controllers with respect to the current tracking error.

(28) $$\left\{\begin{array}{l} u_{d}=k_{dp}(0-i_{d})+k_{di}\int_{0}^{t}(0-i_{d})d\tau \\ u_{q}=k_{qp}(i_{q}^{*}-i_{q})+k_{qi}\int_{0}^{t}(i_{q}^{*}-i_{q})d\tau \end{array}\right.$$

where $i_{q}$ denotes the output of q-axis voltage equation. There are four ELM nodes, parameters $c_{i},v_{i}$ are randomly generated, and the desired angular velocity is $\omega_{r}^{*}=100$ rad/s. The motor is initiated with a 50 N·m load, followed by a sudden impact load of 50 N·m within 0.2 s, before returning to the initial 50 N m load thereafter. Taking into account Δχ, Δη, and Δγ as perturbations of the system parameters, the control parameters are set as

$$\begin{array}{l} a_{1}=1.01,\;b_{1}=11.01;\;p_{1}=0.2,\;T_{s1}=0.01,\;a_{2}=1.10,\;b_{2}=10.01,\\ p_{2}=0.3,\;\gamma =0.01,\;k_{dp}=10,\;k_{di}=15,\;k_{qp}=11,k_{qi}=10 \end{array}$$

### 5.2. A Comparative Simulation Under Different Pdt

Assuming that the CPS is subject to external malicious cyber-attacks, denoted as

$$\kappa_{\mathrm{Dos}}(t)=0.001(3+e^{0.1t}),\;\lambda_{\mathrm{FDI}}(t)=0.01\cos^{2}(3t)$$

**Figure 4:**
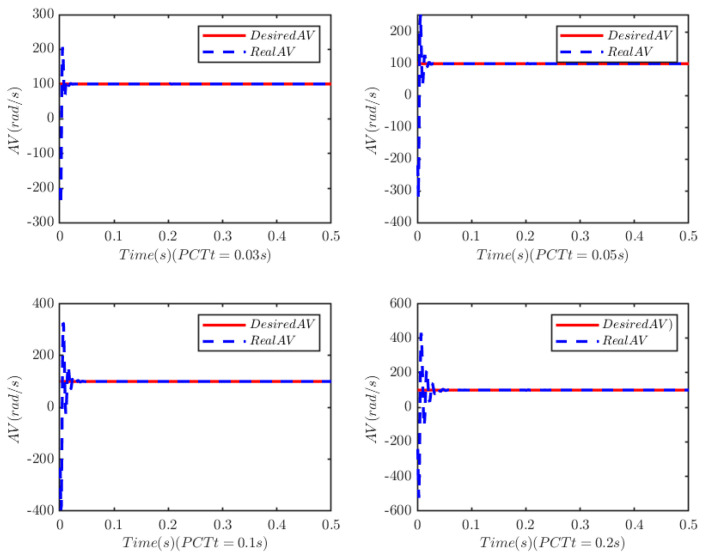
Angular velocity tracking for CPS.

**Figure 5:**
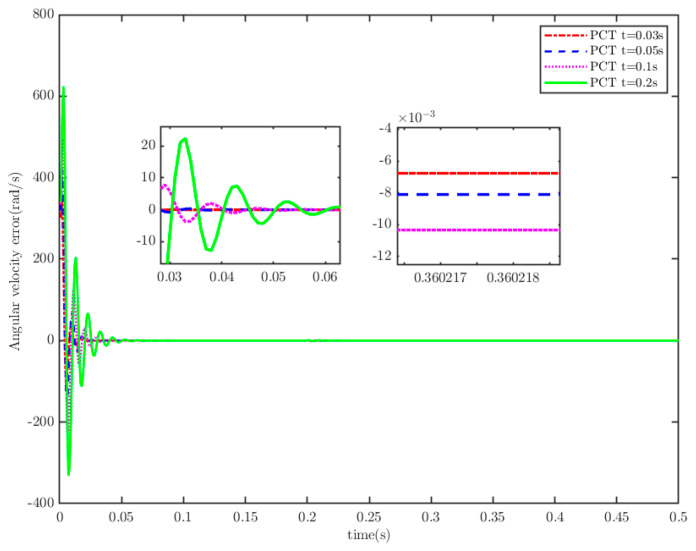
Angular velocity tracking error for CPS.

**Figure 6:**
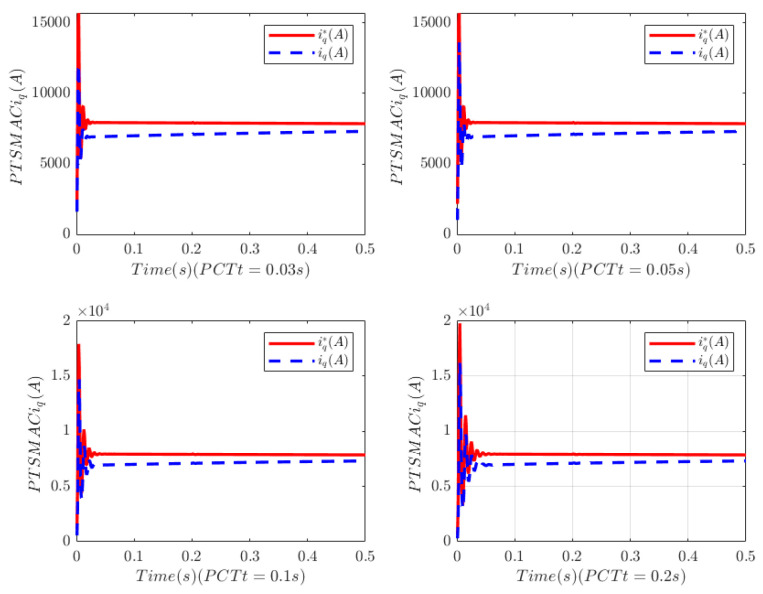
PMSM current tracking schematic.

The load of the PMSM is $T_{L}=\left\{\begin{array}{cc} 100 & t=0.2\\ 50 & t\neq 0.2 \end{array}\right.$. To verify the convergence time of the TTEor of the closed-loop CPS, which can be set in advance, the convergence time of the controller is set as $T_{s2}=0.03\;s,\;0.05\;s,\;0.1\;s,\;0.2\;s$, respectively. Figure 4 and Figure 5 depict the efficacy of the novel sliding-mode adaptive controller formulated in this study, demonstrating its robustness against network attacks and parameter disturbances. Specifically, Figure 5 shows that the TTor for the PMSM in CPS is capable of converging to zero within a predetermined timeframe, despite such adversities. Moreover, convergence accuracy can attain a value of $7\;\times \;10^{-3}$. The smaller the predefined convergence time, the lesser the oscillation of the PMSM angular velocity tracking error, and the greater the convergence precision. This confirms the efficiency of the controller proposed in this paper, indicating that it can effectively mitigate external cyber-attacks. Additionally, by adjusting the convergence time in the controller, the PMSM’s TTEor in the CPS can converge to zero within the PDT. Figure 6 portrays the tracking effect between the output current ($i_{q}$) of the PMSM’s voltage equation and the control input current ($i_{q}^{*}$) of the PMSM’s mechanical motion equation.

Figure 6 illustrates that at the initial stage of control, the control input current in the PMSM’s mechanical motion equation exhibits significant and erratic oscillations, with the maximum input current reaching $1.5\;\times \;10^{4}$ A. Subsequently, the control input current decreases rapidly. There is a deviation between the PMSM’s output current $i_{q}$ and the reference current $i_{q}^{*}$, and the tracking accuracy can be improved by adjusting the control gain of the PI controller based on the current tracking error.

### 5.3. Anti-Interference Robust Analysis

**Figure 7:**
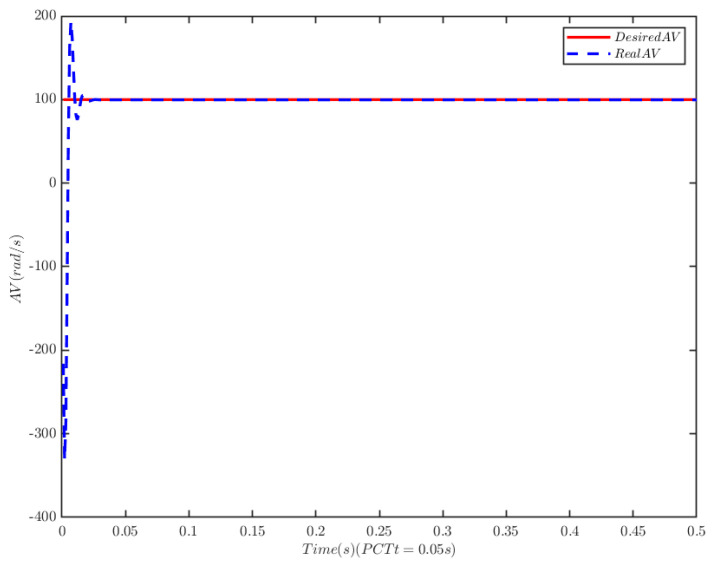
Angular velocity tracking for CPS.

**Figure 8:**
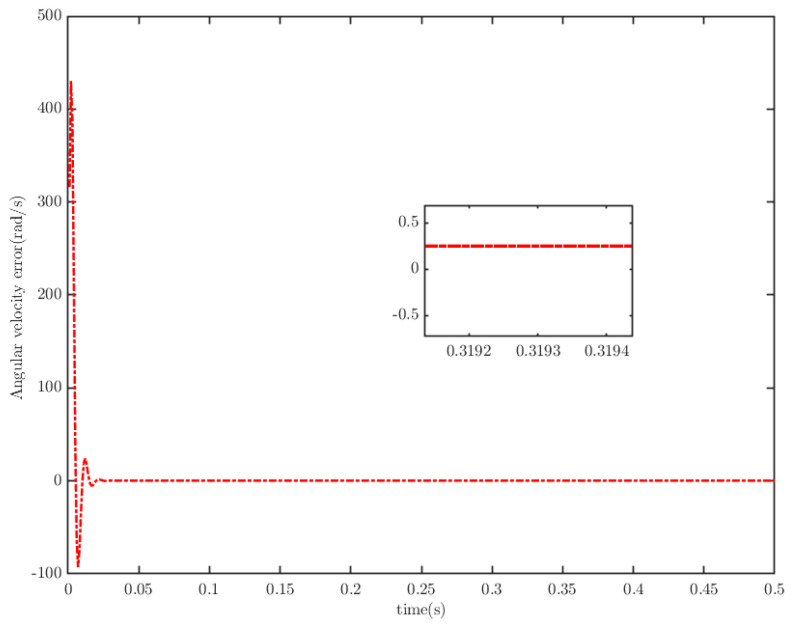
Angular velocity tracking error for CPS.

**Figure 9:**
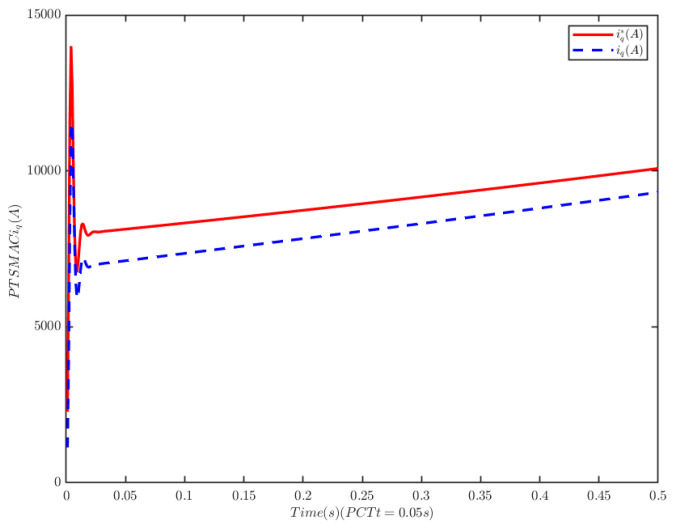
PMSM current tracking schematic.

To verify the anti-interference ability of the novel controller, we assume the malicious cyber-attacks is $\kappa_{\mathrm{Dos}}(t)=0.001(3+e^{0.1t}),\;\lambda_{\mathrm{FDI}}(t)=0.01\cos^{2}(3t)$ and the time-varying load of the PMSM is $T_{L}=50e^{0.5t}$. Under the same control parameters, the simulation results are shown in Figure 7, Figure 8 and Figure 9.

The simulation results demonstrate that when the load of the PMSM increases over time, the TTEor of the PMSM-based CPS can still converge to zero with high accuracy within the PDT. This verifies that the proposed algorithm exhibits strong anti-interference capability.

### 5.4. Performance Comparison and Simulation Analysis of Different Controllers

Considering the presence of unknown disturbances in the physical model of CPS, the following extended state observer (ESO) can be chosen to estimate d(t),

(29) $$\left\{\begin{array}{l} \upsilon =z_{1}-\omega_{r},{\dot{z}}_{1}=z_{2}+\hat{g}+\chi i_{q}^{*}-\beta_{1}\upsilon \\ {\dot{z}}_{2}=-\beta_{2}\tanh(\beta_{3}\theta ),\dot{\hat{g}}=-\eta_{g}s \end{array}\right.$$

where $z_{2}$ is the estimation of d(t) and $\hat{g}$ is the estimation of g; the following controller-based ESO and the PI controller are chosen for comparative simulation.

(1) Super-twisting SMCler based on ESO (STSMC+ESO) [28].

(30) $$\left\{\begin{array}{l} i_{q}^{*}=\frac{1}{\chi }[c\dot{e}-z_{2}+\varepsilon |s{|}^{1/2}\mathrm{sign}(s)+k\int_{0}^{t}\mathrm{sign}(s)d\tau ]\\ s=\dot{e}(t)+ce(t) \end{array}\right.$$

(2) Backstepping L_2_ gain controller based on ESO (BSLGC+ESO) [29].

(31) $$\left\{\begin{array}{l} e=\theta -\theta^{*},a_{l}=-k_{l1}e-{\dot{\theta }}^{*},\eta_{l}=\omega_{r}-a_{l}\\ i_{q}^{*}=-\frac{1}{\chi }[k_{l2}\eta_{l}+z_{2}-\frac{\gamma_{l}^{2}z_{2}^{2}}{2\eta_{l}}-{\dot{a}}_{l}] \end{array}\right.$$

(3) Sliding-mode speed controller based on ESO (SMSC+ESO) [25].

(32) $$\left\{\begin{array}{c} e(t)=\theta^{*}-\theta ,s=\dot{e}(t)+ce(t),\dot{\hat{g}}=-\eta_{g}s\;\;\;\;\;\;\;\;\eta >0\\ i_{q}^{*}=\frac{1}{\chi }[{\ddot{\theta }}^{*}-\hat{g}-z_{2}+\varepsilon |\omega_{r}{|}^{a}\mathrm{sgn}(s)+k|s{|}^{b\mathrm{sgn}(|s|-1)}s+c\dot{e}] \end{array}\right.$$

(4) PI controller.

(33) $$i_{q}^{*}=k_{p}e+k_{i}\int_{0}^{\tau }ed\tau$$

The load of the PMSM is $T_{L}=\left\{\begin{array}{cc} 100 & t=0.2\\ 50 & t\neq 0.2 \end{array}\right.$. Three cyber-attack environments are selected for numerical simulation, respectively, as follows:

**Case 1: Weak cyber-attack**

External malicious cyber-attacks can be described as

$$\kappa_{\mathrm{Dos}}(t)=\left\{\begin{array}{cc} 0.001(3+e^{0.1t}) & t\geq 0.01\\ 1 & 0\leq t<0.01 \end{array}\right.,\;\lambda_{\mathrm{FDI}}(t)=\left\{\begin{array}{cc} 0.01\cos^{2}(3t) & t\geq 0.01\\ 0 & 0\leq t<0.01 \end{array}\right.$$

**Figure 10:**
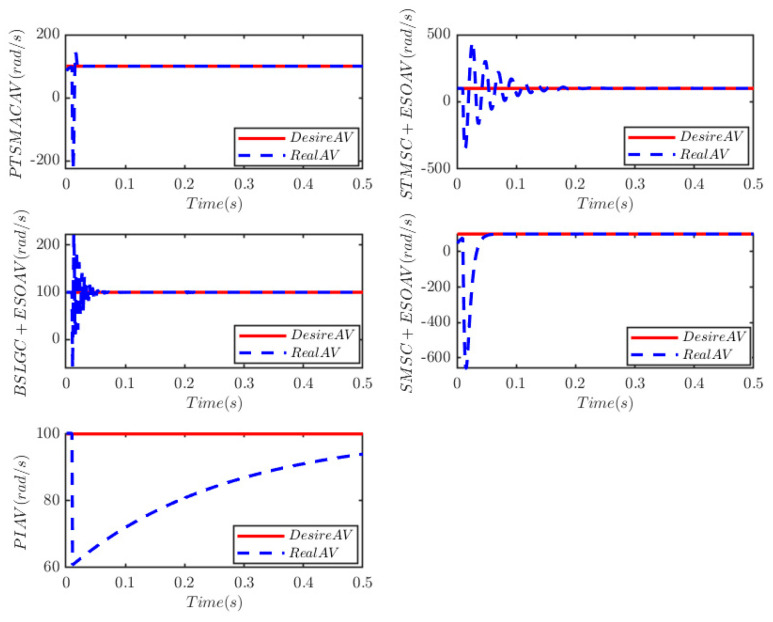
Angular velocity tracking for CPS.

**Figure 11:**
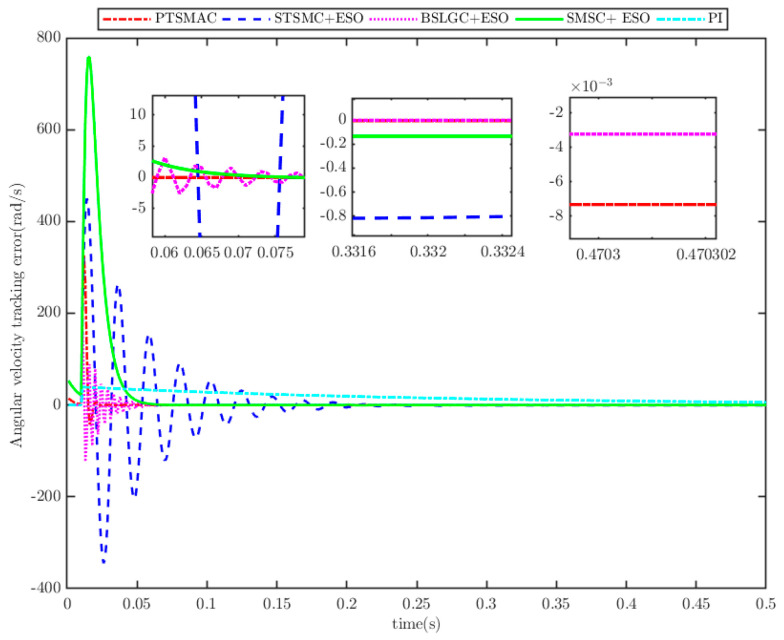
Angular velocity tracking error for CPS.

The results depicted in Figure 10 and Figure 11 demonstrate the remarkable efficacy of the PTSMACler proposed in this paper when the CPS is subjected to a weak cyber-attack.

**Figure 12:**
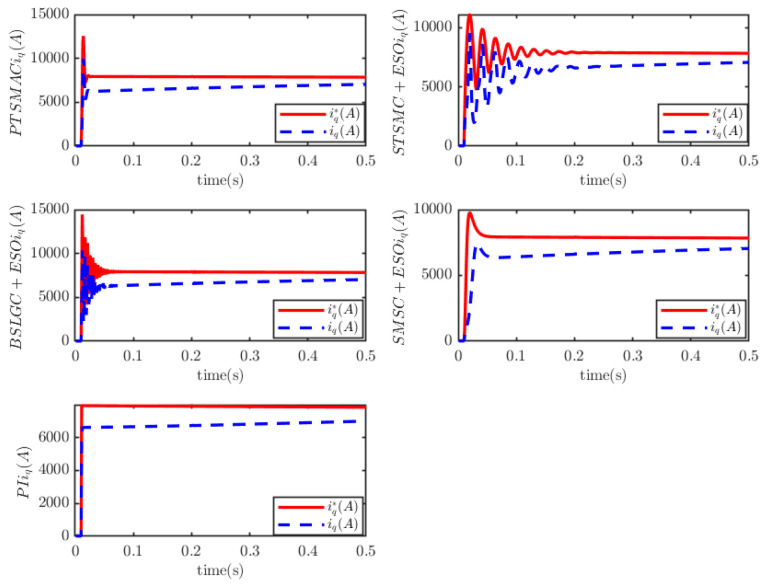
PMSM current-tracking schematic.

It outperforms the other four considered controllers by minimizing the amplitude of the PMSM’s angular velocity tracking error, exhibiting a smoother convergence trajectory, achieving a shorter convergence time, and attaining higher convergence accuracy. Trajectory tracking based on the PI controller exhibits a significant steady-state error. Under the STSMC+ESO and BSLGC+ESO algorithms, the angular velocity tracking error converges with oscillations and requires a longer convergence time. Meanwhile, although the SMSC+ESO algorithm displays comparatively fewer oscillations, it exhibits both a higher maximum tracking error and a larger steady-state tracking error. On the other hand, Figure 12 illustrates that under PTSMAC and SMSC+ESO control, the control input currents of the mechanical dynamics equations exhibit minimal oscillations; however, the current amplitude under PTSMAC is significantly smaller than that under SMSC+ESO control. Conversely, under STSMC+ESO and BSLGC+ESO controls, the control input currents exhibit obvious oscillatory behavior with larger amplitudes. These simulation results unequivocally demonstrate the exceptional performance of the proposed controller against weak cyber-attacks.

**Case 2: High-powered cyber-attack**

High-powered external malicious cyber-attacks can be described as

$$\kappa_{\mathrm{Dos}}(t)=\left\{\begin{array}{cc} 10(3+e^{0.1t}) & t\geq 0.01\\ 1 & 0\leq t<0.01 \end{array}\right.,\;\lambda_{\mathrm{FDI}}(t)=\left\{\begin{array}{cc} 0.1\cos^{2}(3t) & t\geq 0.01\\ 0 & 0\leq t<0.01 \end{array}\right.$$

**Figure 13:**
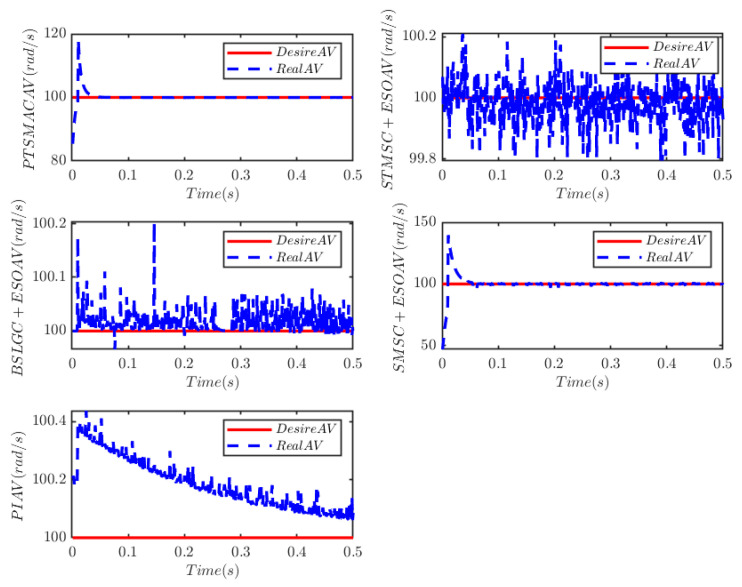
Angular velocity tracking for CPS.

**Figure 14:**
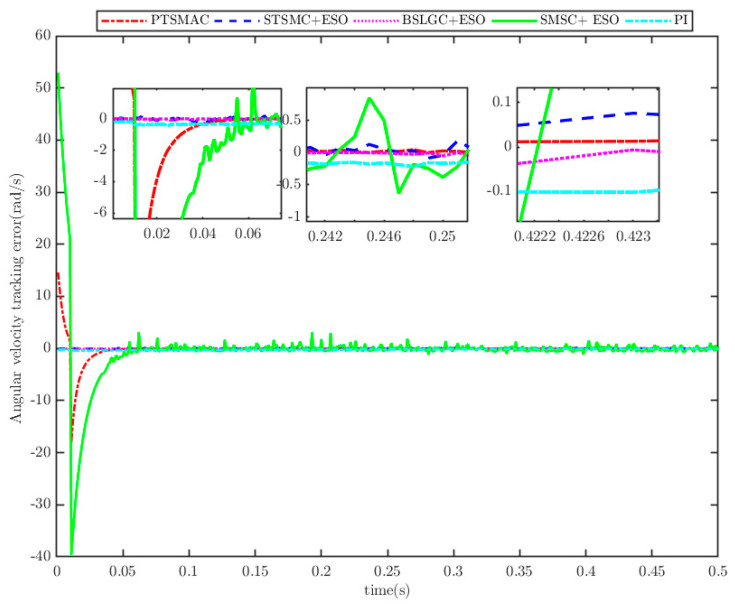
Angular velocity tracking error for CPS.

**Figure 15:**
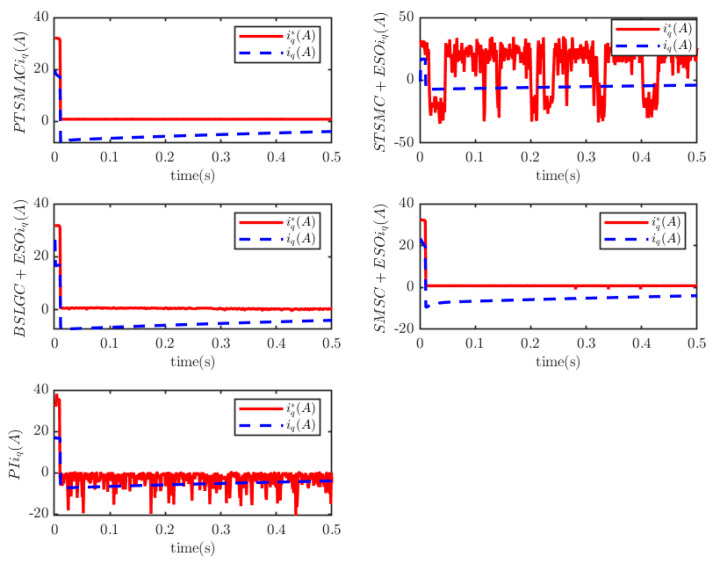
PMSM current tracking schematic.

The results depicted in Figure 13 and Figure 14 demonstrate the remarkable resilience of the PTSMAC, as it successfully drives the angular velocity of the PMSM to zero within a mere 0.05 s, even when subjected to potent multiplicative and additive cyber-attacks. Notably, this convergence is achieved with an impeccably smooth trajectory. These findings unequivocally establish the efficacy of the PTSMAC under high-intensity cyber-attacks. Among its counterparts, the PI, STSMC+ESO, and BSLGC+ESO controllers exhibit rapid angular velocity tracking convergence with near-zero convergence times. However, it is worth mentioning that the SMSC+ESO controller lags significantly in performance, displaying larger convergence errors and more pronounced oscillations in its trajectory. Furthermore, the SMSC+ESO controller also exhibits the longest convergence time among all controllers. Figure 15 visually presents the control input current for each controller employed in this study. It is noteworthy that neither the PTSMAC nor the SMSC+ESO controller induces any oscillations in their respective input currents; however, when the PTSMAC is utilized, the current amplitude is minimized to the smallest possible value. Conversely, under the control of STSMC+ESO and PI, the input current signal exhibits a sharp fluctuation pattern, whereas BSLGC+ESO manages to keep such oscillations relatively subdued.

**Case 3: High-powered cyber-attack**

Other high-powered external malicious cyber-attacks can be described as follows:

$$\kappa_{\mathrm{Dos}}(t)=\left\{\begin{array}{cc} 0.10(3+e^{0.1t}) & t\geq 0.01\\ 1 & 0\leq t<0.01 \end{array}\right.,\;\lambda_{\mathrm{FDI}}(t)=\left\{\begin{array}{cc} 1\cos^{2}(3t) & t\geq 0.01\\ 0 & 0\leq t<0.01 \end{array}\right.$$

**Figure 16:**
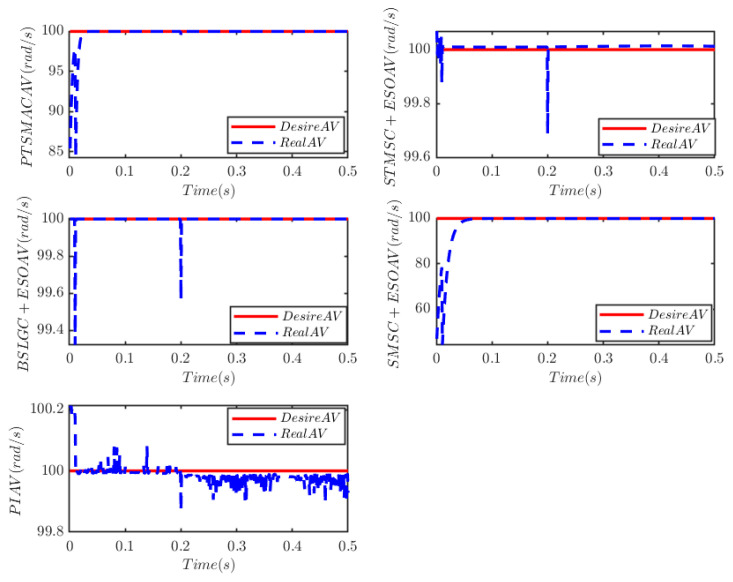
Angular velocity tracking for CPS.

**Figure 17:**
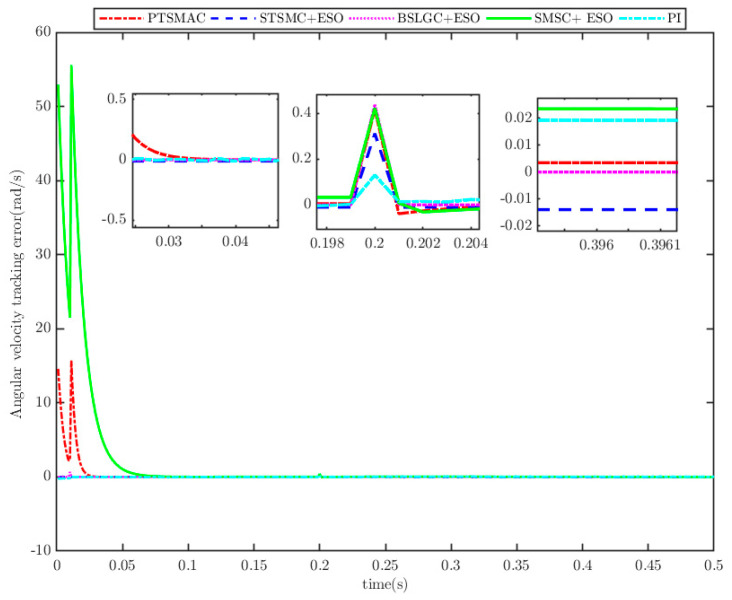
Angular velocity tracking error for CPS.

**Figure 18:**
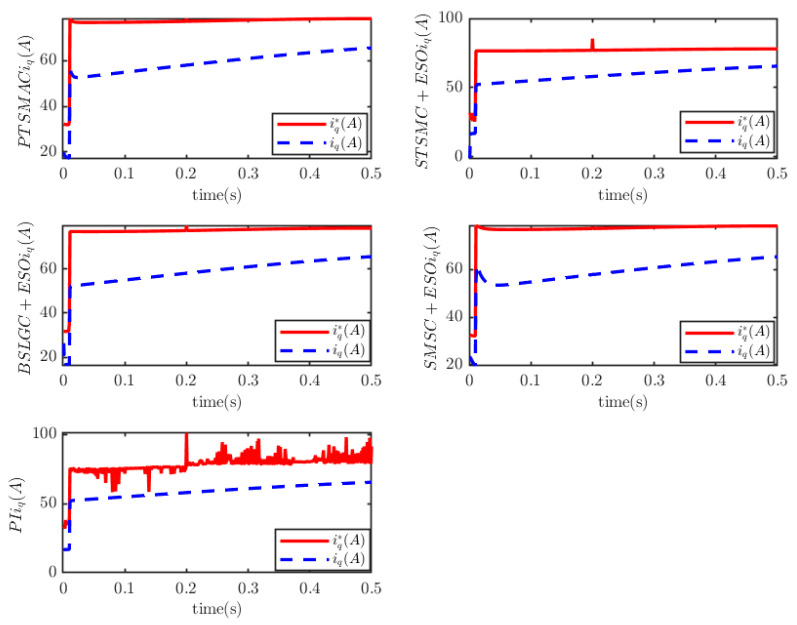
PMSM current tracking schematic.

The simulation results depicted in in Figure 16, Figure 17 and Figure 18 demonstratethat the PMSM’s angular velocity tracking error remains consistently smooth and free of chattering across all five controllers. This holds true even when weak multiplicative and strong additive attacks are launched against the network layer of the CPS. Among these controllers, the PI controller exhibits a significant vibration amplitude in the tracking error; the SMSC+ESO controller demonstrates a remarkably prolonged convergence time for the PMSM’s angular velocity tracking error; while the STSMC+ESO and BSLGC+ESO controllers showcase swift convergence times approaching zero. In stark contrast, the PTSMAC ensures that the PMSM’s angular velocity tracking error converges in less than 0.05 s, thereby meeting the PDT requirement with utmost efficiency. Notably, among these five controllers, the PTSMAC-based control yields the smallest input current amplitude.

The convergence time (CT), steady-state error (SSE), and comprehensive energy consumption (CEC) of the five controllers are listed in Table 3. The results demonstrate that the PTSMAC outperforms the other three controllers, particularly in terms of convergence speed, tracking accuracy, and transient performance. Moreover, the convergence time of the proposed controller is user-definable, whereas the convergence times of the other three controllers cannot be fully preconfigured. Consequently, the proposed PTSMAC offers numerous advantages, including superior control performance and flexible convergence time configuration.

### 5.5. Sensitivity Analysis of ELM Parameters

**Figure 19:**
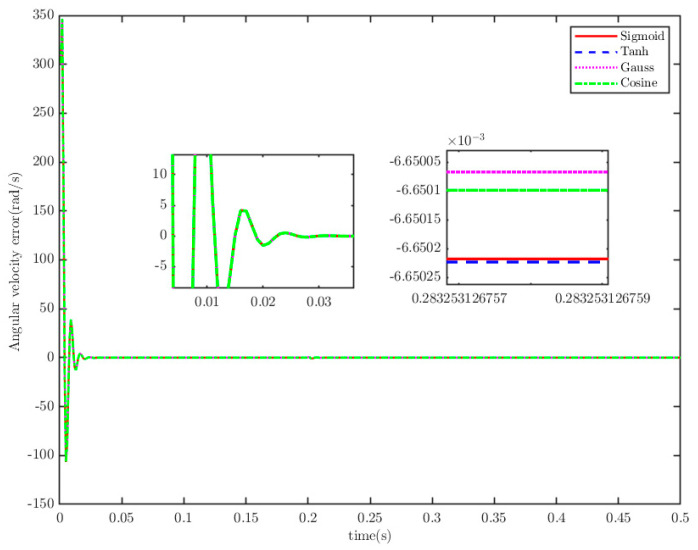
Angular velocity tracking error.

**Figure 20:**
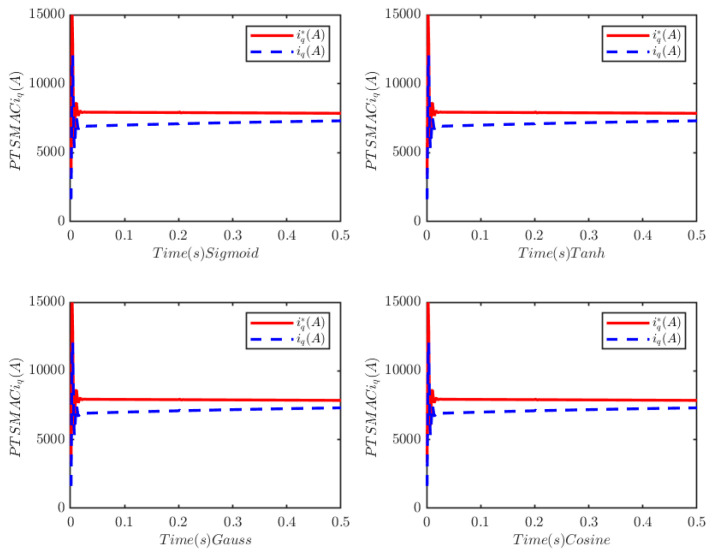
PMSM current tracking schematic.

To analyze the influence of the type of activation function and the number of nodes of the neural network on the control effect, the number of nodes is set to four, and the activation functions are set to the sigmoid function (Sigmoid): Φw(ξ)=(1+exp(−VwξT+bw))−1, hyperbolic tangent function (Tanh): Φw(ξ)=tanh(VwξT+bw), Gauss function (Gauss): $\Phi_{w}(\xi )=\exp(-||\xi -V_{w}|{|}^{2}/b_{w}^{2})$, and cosine Fourier basis function (Cosine): Φw(ξ)=cos (VwξT+bw), respectively, with other parameters unchanged. The numerical simulation results are shown in Figure 19 and Figure 20. It can be observed from the simulation results that the type of activation function does not affect the control performance of the cyber–physical system (CPS).

**Figure 21:**
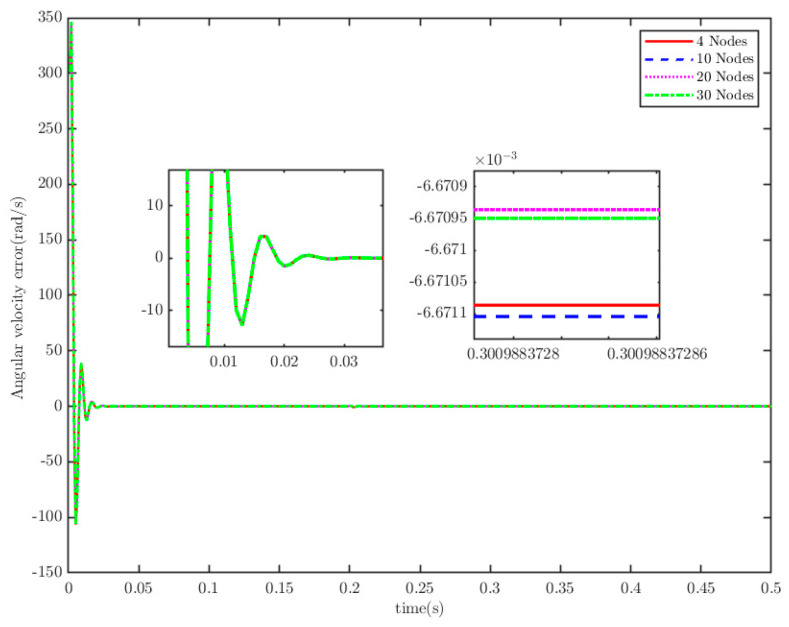
Angular velocity tracking error.

**Figure 22:**
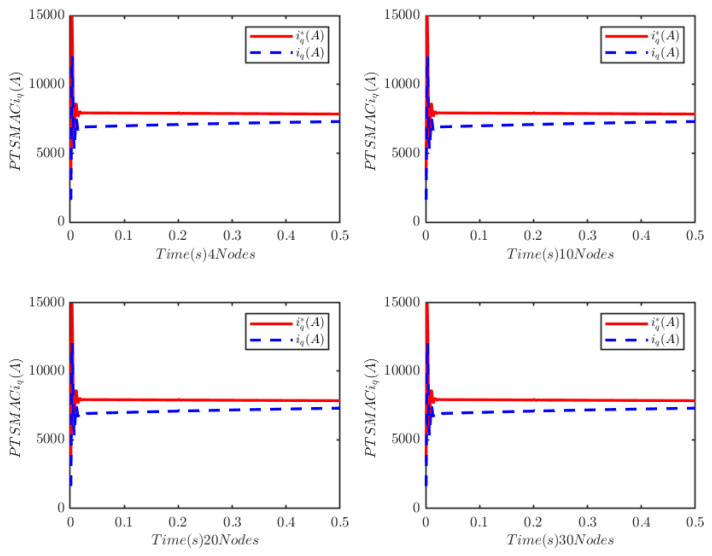
PMSM current-tracking schematic.

Similarly, the activation function is fixed as the sigmoid function, and the number of nodes of the ELM is set to 4, 10, 20, and 30, respectively, for numerical simulations. The simulation results are presented in Figure 21 and Figure 22. These results indicate that the number of nodes also has no impact on the control performance of the CPS.

## 6. Conclusions

We have investigated the trajectory tracking control problem of CPS based on PMSM, focusing on the impacts of malicious cyber-attacks at the cyber layer, as well as parameter perturbations and external loads at the physical layer. A model-free and disturbance-independent sliding-mode adaptive safety control strategy is proposed, which only requires information about the motor’s angular velocity tracking error and supports predefined convergence time. Numerical simulations confirm that this safety control strategy can resist malicious cyber-attacks and exhibits strong anti-interference capability.

A novel PreTC Lyapunov stability criterion is proposed, and an SMS with predefined convergence time is designed, enriching the theoretical framework of PreTC. The upper bound of the predefined convergence time is not affected by the initial system state or controller parameters, which not only enhances the degree of freedom in the design of time-optimal control algorithms but also renders the convergence time of the closed-loop system controllable. By using an ELM to approximate the aggregated term composed of the PMSM model and external disturbances, a novel sliding-mode adaptive controller is designed for PMSM-based CPS under cyber-attacks. This controller can effectively mitigate the adverse effects of malicious cyber-attacks, system parameter perturbations, and external heavy loads on the CPS, improve the robustness of the PMSM-based CPS, and rapidly achieve high-precision angular velocity tracking—thus effectively expanding the application scenarios of PMSM-based CPS. Through comparative simulations of four safety control strategies under weak and strong cyber-attacks, it is demonstrated that the proposed algorithm offers multiple advantages, including smaller TTEor, a smoother convergence trajectory, shortened convergence time, and higher convergence accuracy.

The next step will involve constructing a physical model to verify the engineering application effect of the proposed algorithm through physical simulation experiments, considering actual constraints and disturbance issues. Additionally, while this paper uses an ELM to approximate the aggregated term, the output weights of the ELM lack convergence, which cannot guarantee the accurate estimation of the aggregated term by the ELM and may increase control energy consumption. In future research, designing an ELM output weight update law with convergence characteristics and completing the corresponding stability theoretical proof will be the key direction for improvement.

## Figures and Tables

**Table 1:** Stability criterion and convergence time.

	Lyapunov Candidate	Upper of Convergence Time
Asymptotically stable system	$\dot{\sigma }(x(t))<0$	$T(x_{0})\leq T_{\max}=\infty$
Finite-time stability	$\dot{\sigma }(x)\leq -\lambda_{1}\sigma (x)-\lambda_{2}\sigma {\left(x\right)}^{\alpha }$	$T(x_{0})\leq T_{\max}=\frac{1}{\lambda_{1}(1-\alpha )}\ln\frac{\lambda_{1}\sigma {\left(x_{0}\right)}^{1-\alpha }+\lambda_{2}}{\lambda_{2}}$
Fixed-time stability	$\dot{\sigma }(x)<-\alpha_{1}\sigma {\left(x\right)}^{\beta_{1}}-\alpha_{2}\sigma^{\beta_{2}}(x)$	$T(x_{0})\leq T_{\max}=\frac{1}{\alpha_{1}(1-\beta_{1})}+\frac{1}{\alpha_{2}(\beta_{2}-1)}$
Predefined-time stability	$\dot{\sigma }(x)\leq \frac{-\pi (a_{2}\sigma {\left(x\right)}^{1-p_{2}}+b_{2}\sigma {\left(x\right)}^{1+p_{2}})}{2p_{2}T_{s2}\sqrt{a_{2}b_{2}}}$	$T(x_{0})\leq T_{\max}=T_{s2}$

**Table 2:** Parameters of PMSM.

Parametric	Notation	Value	Unit
Number of motor poles	p	6	/
Resistance	R	1.55	Ω
Stator inductance	L	5 × 10^−3^	H
d-axis inductor	$L_{d}$	6.71 × 10^−3^	H
q-axis inductor	$L_{q}$	6.71 × 10^−3^	H
Permanent magnet chain	$\psi_{f}$	0.175	Wb
Inertia moment	J	0.0002	kg·m^2^
Damping factor	B	0.0003	N·m·s
Parametric perturbance	Δχ	20 sin(t)	/
	Δη	10 sin(t)	/
	Δγ	10 sin(t)	/

**Table 3:** Performance comparison under different controllers.

Controller	Case 1			Case 2			Case 3		
	CT	SSE	CEC	CT	SSE	CEC	CT	SSE	CEC
PTSMAC	0.030 s	0.007	3.8732 × 10^6^	0.05 s	0.006	0.0709 × 10^4^	0.042 s	0.025	0.3332 × 10^4^
STSMC+ESO	0.320 s	0.630	3.8668 × 10^6^	0.01 s	0.20	1.0243 × 10^4^	0.016 s	0.015	3.8167 × 10^4^
BSLGC+ESO	0.102 s	0.003	3.8669 × 10^6^	0.01 s	0.10	0.0569 × 10^4^	0.012 s	0.001	3.8331 × 10^4^
SMSC+ESO	0.068 s	0.140	3.8684 × 10^6^	0.06 s	3.00	0.0719 × 10^4^	0.084 s	0.037	3.8349 × 10^4^
PI	0.450 s	16.800	3.8646 × 10^6^	0.01 s	0.4	17.7741 × 10^4^	0.013 s	0.027	3.9071 × 10^4^

## Data Availability

The authors confirm that the data supporting the findings of this study are available within the article.

## Abbreviation

ELM	Extreme learning machine	SMS	Sliding-mode surface
CPS	Cyber–physical system	DoS	Denial-of-service
FDI	False data injection	PreTC	Predefined-time convergence
PMSM	Permanent magnet synchronous motor	TTEor	Trajectory tracking error
PDT	Predefined time	NLS	Nonlinear system
SMC	Sliding-mode control	UB	Upper bound

## References

[1] Ding Y., Yin C.W., Riaz S. (2025). A novel PT-SMC based resilient control of cyber-physical robotic system under malicious-threats. ISA Trans..

[2] Ge X., Han Q.L., Zhong M., Zhang X.M. (2019). Distributed Krein space-based attack detection over sensor networks under deception attacks. Automatica.

[3] Deng R., Xiao G., Lu R. (2015). Defending against false data injection attacks on power system state estimation. IEEE Trans. Ind. Inform..

[4] Rawat D.B., Bajracharya C. (2015). Detection of false data injection attacks in smart grid communication systems. IEEE Signal Process. Lett..

[5] Li B., Lu R., Xiao G., Li T., Choo K.-K.R. (2022). Detection of False Data Injection Attacks on Smart Grids: A Resilience-Enhanced Scheme. IEEE Trans. Power Syst..

[6] Xie C.H., Yang G.H. (2018). Observer-based attack-resilient control for linear systems against FDI attacks on communication links from controller to actuators. Int. J. Robust Nonlinear Control.

[7] Li T., Wang L., Li Z. (2023). Observer-based adaptive event-triggered neural tracking control for nonlinear cyber-physical systems with incomplete measurements. Int. J. Adapt. Control Signal Process..

[8] Su L., Ye D., Zhao X.-G. (2021). Distributed secure state estimation for cyber-physical systems against replay attacks via multisensor method. IEEE Syst. J..

[9] Meng Q., Kasis A., Polycarpou M. (2025). Sliding Mode Control for Robustness in Networked Switched Systems Under Denial-of-Service Attacks. IEEE Trans. Autom. Control.

[10] Yin C. (2025). Model-free sliding mode resilient control of cyber-physical system based on reinforcement learning. Appl. Intell..

[11] Zhang W., Du H., Zhu W. (2021). Finite-time speed sensorless control of permanent magnet synchronous motor based on generalized super-twisting algorithm. Control Theory Appl..

[12] Chen Z., Lin Z., Jia H. (2021). Finite-time control for permanent magnet synchronous motor with prescribed performance. Control Theory Appl..

[13] Li S., Zhou M., Yu X. (2012). Design and implementation of terminal sliding mode control method for PMSM speed regulation system. IEEE Trans. Ind. Inform..

[14] Feng Y., Yu X., Han F. (2012). High-order terminal sliding-mode observer for parameter estimation of a permanent-magnet synchronous motor. IEEE Trans. Ind. Electron..

[15] Farbood M., Echreshavi Z., Mobayen S., Skruch P. (2025). Resilient event-triggered observer-based control of uncertain nonlinear fuzzy systems subject to unknown inputs. IEEE Access.

[16] Echreshavi Z., Farbood M., Shasadeghi M., Mobayen S. (2024). Robust event-triggered data-driven control subject to control constraints. Soft Comput..

[17] Echreshavi Z., Farbood M., Shasadeghi M., Mobayen S. (2025). Data-driven adaptive event-triggered terminal sliding mode control for nonlinear systems with prescribed performance. Int. J. Robust Nonlinear Control.

[18] Yang Z., Zhao Y., Gao F. (2021). Fixed-time stabilization for a wheeled mobile robot with actuator dead-zones. IAENG Int. J. Comput. Sci..

[19] Xu P. (2020). Fixed time control of dynamic positioning ship with unknown interference. Open J. Appl. Sci..

[20] Wang L., Du H., Zhang W., Wu D., Zhu W. (2020). Implementation of integral fixed-time sliding mode controller for speed regulation of PMSM servo system. Nonlinear Dyn..

[21] Yin C., Ding Y., Sun H. (2025). Nonsingular Fast Predefined Time Convergence Sliding Mode Control for Construction Robot. ISA Trans..

[22] Chavoshi H., Sedgh A., Khaloozadeh H. (2025). Resilience PI controller design for mitigating weak denial-of-service attacks in cyber-physical systems. IET Cyber-Phys. Syst. Theory Appl..

[23] Yin C., Yi P., Bu X. (2025). Neural network-based guaranteed performance sliding mode security control of nonlinear cyber-physical systems. Eng. Appl. Artif. Intell..

[24] Munoz-Vazquez A.J., Sánchez-Torres J.D., Jimenez-Rodriguez E., Loukianov A.G. (2019). Predefined-time robust stabilization of robotic manipulators. IEEE/ASME Trans. Mechatron..

[25] Wang Y., Feng Y., Zhang X., Liang J. (2020). A new reaching law for anti-disturbance sliding-mode control of PMSM speed regulation system. IEEE Trans. Power Electron..

[26] Raoufi M., Habibi H., Yazdani A., Wang H. (2022). Robust prescribed trajectory tracking control of a robot manipulator using adaptive finite-time sliding mode and extreme learning machine method. Robotics.

[27] Huang G.B., Zhu Q.Y., Siew C.K. (2006). Extreme learning machine: Theory and applications. Neurocomputing.

[28] Levant A. (1993). Sliding order and sliding accuracy in sliding mode control. Int. J. Control.

[29] Forbes J.R. (2017). L2-gain and passivity techniques in nonlinear control. IEEE Control Syst. Mag..

